# Anticoagulation Strategies for Atrial Fibrillation in CKD Stage G5 and Dialysis Patients: An Updated Scoping Review

**DOI:** 10.31083/RCM26736

**Published:** 2025-03-05

**Authors:** Heitor Martins de Oliveira, Lorrany Pereira Barros, Maria Clara Azzi Vaz de Campos, Rafael Ferreira Daher, Gil Batista Gonçalves, Mateus Teodoro Sequeira, Silvia Marçal Botelho, Antonio da Silva Menezes Junior

**Affiliations:** ^1^Faculdade de Medicina, Pontifícia Universidade Católica de Goiás, 74605-010 Goiânia, Goiás, Brazil; ^2^Departamento de Clínica Médica, Faculdade de Medicina da Universidade Federal de Goiás, 74605-050 Goiânia, Goiás, Brazil

**Keywords:** atrial fibrillation, chronic kidney disease, anticoagulation, direct oral anticoagulants, dialysis

## Abstract

Clinical trials of direct oral anticoagulants (DOACs) often exclude patients with advanced chronic kidney disease (CKD), creating uncertainty regarding their safety and efficacy compared with warfarin. This study addresses this gap by providing key insights into anticoagulation in this high-risk population. This study evaluated the effectiveness and safety of DOACs compared to warfarin and no anticoagulation therapy in atrial fibrillation (AF) patients with CKD stage G5 or on dialysis. This scoping review followed a six-stage framework and Preferred Reporting Items for Systematic Reviews and Meta-Analyses (PRISMA) guidelines. An exhaustive search of four databases identified relevant papers published through August 2024. The data extraction process was conducted independently, with subsequent qualitative and quantitative analyses conducted. Among the 33 studies included in the final analysis, DOACs, particularly apixaban, were associated with a 20–30% decreased major bleeding risk compared to warfarin. Stroke incidence was comparable between DOACs and vitamin K antagonists (VKAs), with apixaban showing improved prevention in severe CKD. Observational studies reported slightly lower mortality rates with DOACs, particularly apixaban, including fewer cardiovascular-related deaths than with VKAs. DOACs, particularly apixaban and rivaroxaban, demonstrate a favorable safety profile compared to warfarin, but show inconsistent evidence in balancing thromboembolic prevention and bleeding risks in patients with AF and CKD stage G5 or on dialysis. Future studies should focus on optimizing dosing strategies and evaluating long-term safety and efficacy.

## 1. Introduction

Atrial fibrillation (AF) is the most common sustained cardiac arrhythmia, affecting 1–2% of the population, with increasing incidence due to aging populations and the rising prevalence of risk factors such as hypertension, diabetes, and obesity [1]. As a significant public health challenge, AF increases the risk of adverse outcomes, including ischemic stroke, systemic embolism, heart failure, and mortality. Notably, ischemic stroke remains a leading complication of AF, associated with increased morbidity, mortality, and long-term disability. Stroke prevention is therefore a cornerstone of AF management, aiming to reduce the disease burden on both individuals and global healthcare systems [2, 3, 4].

**Fig. 1.**
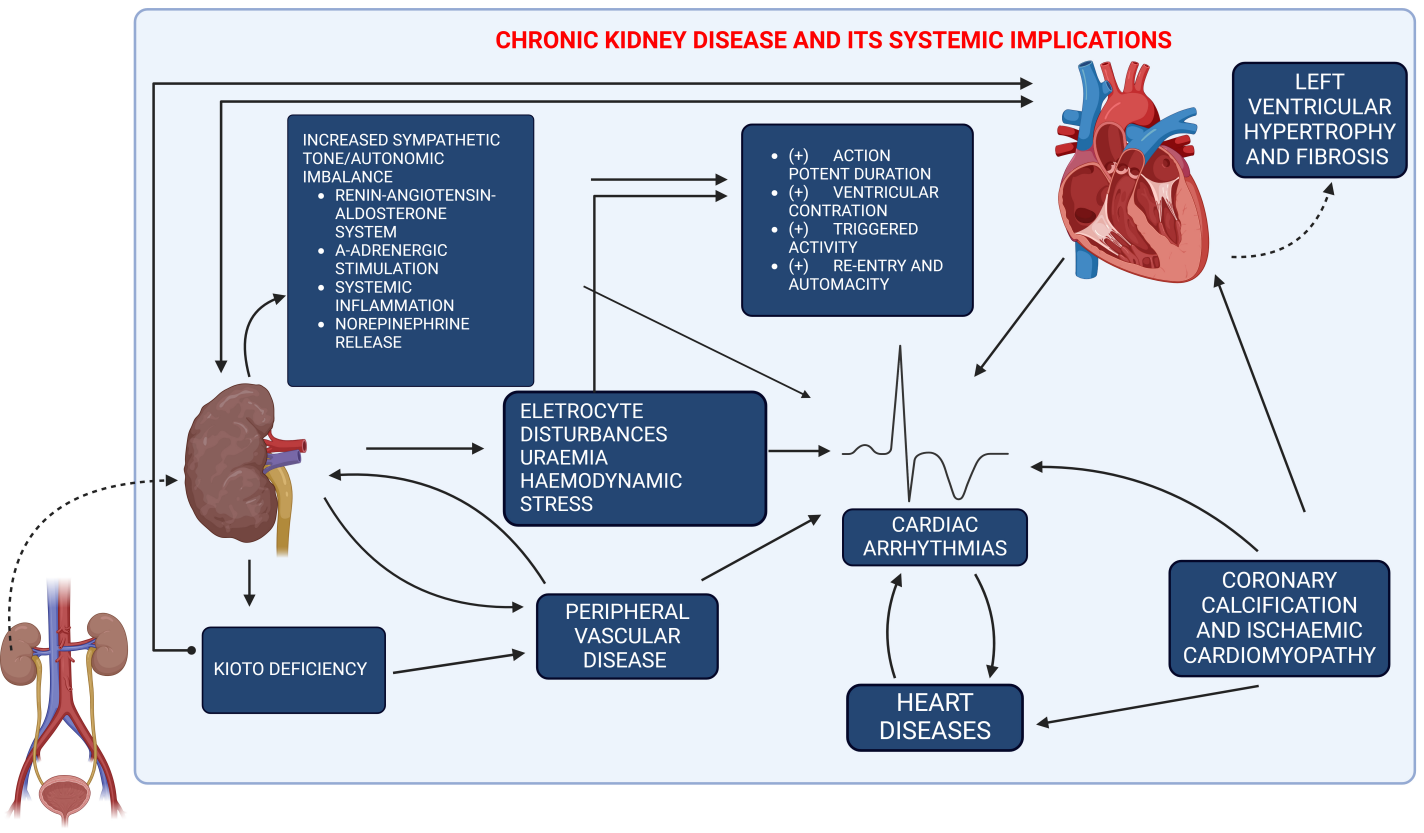
**Pathophysiological mechanisms linking chronic kidney disease and atrial fibrillation**. This figure illustrates the key mechanisms that contribute to the bidirectional relationship between chronic kidney disease (CKD) and atrial fibrillation (AF). CKD promotes structural and electrical remodeling of the atria through processes such as left ventricular hypertrophy, increased sympathetic nervous system activation, systemic inflammation, oxidative stress, and endothelial dysfunction. These factors enhance atrial remodeling and arrhythmogenesis, increasing the likelihood of AF. Conversely, AF exacerbates renal dysfunction by impairing renal perfusion and promoting systemic vascular inflammation. Together, these interrelated mechanisms create a vicious cycle that amplifies cardiovascular risk in this patient population.

The interplay between AF and chronic kidney disease (CKD) introduces additional treatment challenges. CKD affects approximately 10% of the global population, with an estimated 850 million individuals experiencing a degree of renal impairment [5]. It is characterized by progressive deterioration of kidney function, often progressing to end-stage renal disease (ESRD) necessitating dialysis or kidney transplantation [6]. CKD exacerbates cardiovascular risk and significantly increases AF prevalence [5, 6, 7, 8, 9, 10], ranging from 15% in mild-to-moderate CKD stages to nearly 40% in ESRD. The pathophysiological mechanisms linking CKD to AF include left ventricular hypertrophy, increased sympathetic nervous system activation, systemic inflammation, and endothelial dysfunction, which collectively promote atrial remodeling and arrhythmogenesis [11] (Fig. 1).

The coexistence of AF and CKD presents substantial challenges in patient management owing to the bidirectional nature of their interactions. CKD accelerates AF progression, leading to higher rates of thromboembolic complications [12]. Conversely, AF exacerbates renal outcomes, creating a cycle of compounded risks. This interplay significantly increases the risk of thromboembolic events, including ischemic stroke and hemorrhagic complications, particularly with anticoagulation therapy [13].

Anticoagulation therapy is a cornerstone of stroke prevention in AF patients. Traditionally, vitamin K antagonists (VKAs), such as warfarin, have been the primary therapeutic option, used as anticoagulants in this population. VKAs reduce stroke risk, their use in CDK patients is limited by substantial challenges [14, 15]. Particularly, of VKAs exhibit high a variability in therapeutic response, necessitating monitoring and dose adjustments, due to dietary influences, drug interactions, and altered pharmacokinetics observed in CKD. Furthermore, VKA therapy in patients with CKD has been associated with an increased risk of bleeding complications and promotion of vascular calcification. This phenomenon exacerbates cardiovascular morbidity and mortality [16].

In recent years, direct oral anticoagulants (DOACs) have emerged as alternatives to VKAs, offering several advantages, including fixed dosing, fewer drug-drug interactions, and a reduced need for monitoring. In the general population with AF, DOACs have demonstrated efficacy comparable to VKAs in preventing stroke and systemic embolism, with a lower risk of major bleeding [17, 18, 19]. However, their utilization in patients with advanced CKD (stage G5) and those on dialysis remains controversial owing to the partial renal excretion of these agents and the exclusion of these high-risk populations from pivotal clinical trials. Consequently, the safety and efficacy of DOACs in this subgroup of patients remains poorly defined [20].

The lack of robust evidence creates significant uncertainty in determining the optimal anticoagulation strategy for patients with AF and CKD stage G5, including those on dialysis. Observational studies have provided preliminary insights, with emerging data suggesting that certain DOACs, particularly apixaban, may offer safer alternatives to VKAs in this population [14, 15, 16, 18, 19]. However, the findings remain inconsistent and questions persist regarding the appropriate dosing, bleeding risks, and long-term outcomes of DOAC therapy in this high-risk group. Additionally, the decision to initiate anticoagulation therapy in dialysis patients with AF is further complicated by the need to balance the high thromboembolic risk associated with AF with the heightened bleeding risk inherent to dialysis [21].

The clinical significance of these challenges is profound, as the population of patients with AF and advanced CKD is expected to grow rapidly. This increase is driven by global demographic aging, the rising prevalence of risk factors such as diabetes and hypertension, and the broader trends of increasing CKD incidence. Therefore, evidence-based guidance is urgently needed to optimize anticoagulation strategies for this high-risk group [20, 21, 22].

This scoping review sought to address this critical gap in the literature by evaluating the safety and efficacy of DOACs compared to VKAs in the absence of other anticoagulation treatments in patients with AF and CKD stage G5 or on dialysis. By synthesizing data from available studies, this review aimed to provide clinicians with insights into the potential benefits and risks of anticoagulation in this complex patient population, ultimately contributing to improved clinical decision-making and patient outcomes.

## 2. Methods

### 2.1 Type of Study

The scoping review structure, initially proposed by Arksey and O’Malley (2005) [23], consists of five distinct steps: First, identifying the research question, identifying relevant studies, study selection, charting the data, and finally, collating, summarizing, and reporting results. In our study, we did not perform any consultation during the sixth step. Furthermore, the Preferred Reporting Items for Systematic Reviews and Meta-Analyses Extension for Scoping Reviews (PRISMA-ScR) checklist helped to perform this review [24]. A scoping review was chosen instead of a meta-analysis to provide a comprehensive overview of the existing literature on the safety and efficacy of anticoagulation therapies in patients with CKD and AF. This approach is particularly suitable for addressing this study’s broad and complex research questions. The goal was to map the available evidence, identify key concepts, and highlight gaps in knowledge within the field, especially given the high heterogeneity among the studies.

### 2.2 Data Search 

**Fig. 2.**
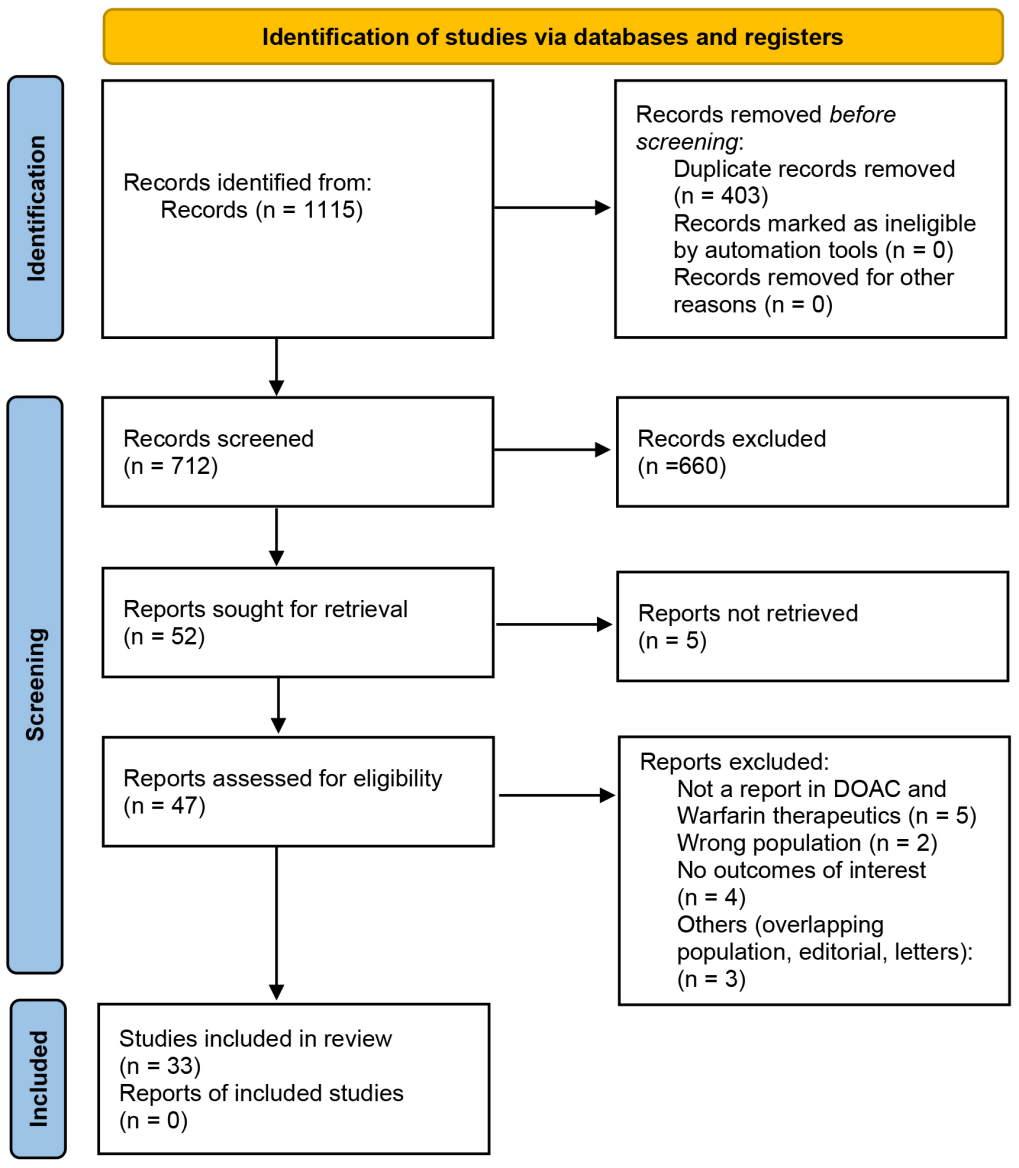
**Study selection flowchart for inclusion in the systematic review**. This flowchart outlines the study selection process for the systematic review, detailing the progression from the initial database search to the final inclusion of studies. DOAC, direct oral anticoagulant.

A preliminary search was performed in PubMed, Embase, Scopus, Web of Science, and Cochrane databases, with no restrictions, using the keywords Medical Subject Headings (MeSH) or Emtree terms discovered in the previous search, as shown in Fig. 2. In addition, searches were undertaken in two gray literature databases (Google Scholar and ProQuest), which were challenging to discover or unpublished. The last search was conducted on August 17, 2024. In addition, references to the related literature and review papers were consulted for further information. This review was registered with the Open Science Framework (DOI registry DOI 10.17605/OSF.IO/Y9DG6).

### 2.3 Selecting Studies – Eligibility Criteria 

This study included studies on anticoagulation while comparing DOACs to VKAs and focusing on patients with stage 5 CKD or dialysis for CKD stage G5. The inclusion criteria also covered studies published in English. The exclusion criteria were non-original research, patients with valvular atrial fibrillation, papers with insufficient data, and articles written in languages other than English. The reviewers reviewed the titles and abstracts of the documents individually using the criteria outlined above, and the surviving publications were subjected to a full-text review to guarantee their eligibility for the final data analysis. Any disagreements were settled via conversation or if necessary, with the assistance of a third reviewer.

### 2.4 Data Collection and Graphing

Two reviewers separately recorded the data and plotted them on pre-designed charts using Excel software. The charts display the first author’s name, publication year, origin, study type, anticoagulant used, number of patients, sex (%), age (mean ± SD), expression levels, and the main findings.

### 2.5 Synthesizing and Presenting Results

Quantitative and qualitative assessments were also performed. A descriptive numerical summary of the features of the publications was provided for quantitative analysis. For qualitative analysis, we conducted a narrative evaluation of the present data in response to our previously established research question, while concentrating on the importance of the results in a broader context.

## 3. Results

A comprehensive search of multiple databases identified 1115 records related to DOAC and Warfarin therapy. Following the removal of 403 duplicates, 712 articles underwent title and abstract screening, resulting in the exclusion of 660 articles. After assessing the remaining 52 full-text articles, 19 were excluded due to unrelatedness, inappropriate study populations, or unavailability of full texts. Ultimately, 33 studies met the inclusion criteria, providing a comprehensive overview of the safety and efficacy of DOACs compared with warfarin in patients with AF and CKD stage G5 undergoing dialysis [25, 26, 27, 28, 29, 30, 31, 32, 33, 34, 35, 36, 37, 38, 39, 40, 41, 42, 43, 44, 45, 46, 47, 48, 49, 50, 51, 52, 53, 54, 55, 56]. The analysis focused on three primary outcomes: major bleeding, stroke, and mortality (**Supplementary Table 1**). The primary outcomes are summarized in Table 1 (Ref. [25, 26, 27, 28, 29, 30, 31, 32, 33, 34, 35, 36, 37, 38, 39, 40, 41, 42, 43, 44, 45, 46, 47, 48, 49, 50, 51, 52, 53, 54, 55, 56]).

**Table 1.** **Key characteristics of the included studies**.

Author/Year	Country	Patients (n)	Female (%)	Age (y)	Follow up (months)	Study	SAH	DM	CHADS2 VASC score	CKD stage (n)	Dialysis (n)	Previous stroke n (%)
Harrington *et al*. (2023) [25]	USA	71,683	37.3	70.6 ± 9.4	23.1	Individual patient-level network meta-analysis	62,863	22,087	4.0 ± 1.5	NR	NR	20,147 (28.1)
Pokorney *et al*. (2022) [26]	USA	154 (apixaban [82], warfarin [72])	36.4	68 ± 10.37	12	RCT	146	89	4.0 ± 1.48	5	154	29 (18.8)
Wetmore *et al*. (2022) [27]	USA	14,899 (apixaban [2382], warfarin [12,517])	38.3	66.2 ± 9.4	NR	Retrospective cohort study	14,199	11,413	4.7 ± 1.7	5	14,899	NR
Sarratt *et al*. (2017) [28]	USA	160 (warfarin [120], apixaban [40])	51.25	68.7 ± 17.9	NR	Retrospective cohort study	NR	NR	5 ± 3.7	5	160	34 (47)
Königsbrügge *et al*. (2017) [29]	Austria	626	36.6	66 ± 14.8	NR	Prospective observational cohort study	576	160	NR	5	626	127 (20.3)
Reinecke *et al*. (2023) [30]	Germany	97 (apixaban [48], VKA [49])	29.9	77 ± 8.7	Apixaba 14.10, VKA 116.75	RCT	NR	NR	5 ± 1.48	5	97	NR
AlTurki *et al*. (2024) [31]	Canada	10,036 (apixaban [2638], varfarina [7398])	54	60 to 74 years (apixaban) and 62 to 71 years (warfarin)	10 to 12	Systematic Review and Meta-Analysis	NR	NR	NR	5	10,036	137 (1.3)
De Vriese *et al*. (2021) [32]	Belgium	90 (rivaroxaban [46], VKA [44])	33.34	80.3 (71.5–84.3)	22 to 54	RCT	NR	62	5 ± 1.48	5	132	40 (30.3)
Kao *et al*. (2024) [33]	Taiwan	184,136 (DOAC 8861 [4.81%], VKA 70,047 [38.04%], not anticoagulated 105,228 [57.15%])	NR	NR	NR	Network meta-analysis	NR	NR	NR	5	NR	621 (0.34)
Chen *et al*. (2021) [34]	Taiwan	25 articles (6 RCTs and 19 observational studies)	NR	NR	NR	Systematic review and meta-analysis	NR	NR	NR	3–5	NR	NR
Kuno *et al*. (2020) [35]	USA, Japan	16 articles (71,877)	NR	68.9	18.0 to 52.8	Systematic review and meta-analysis	NR	NR	NR	5	71,877	NR
Fu *et al*. (2024) [36]	USA	18,208 (apixaban and rivaroxaban [12,488], apixaban [5720])	50.24	78.8 ± 7.7	9.09	Retrospective cohort study	17,832 (warfarin versus apixaban [12,229], rivaroxaban versus apixaban [5603])	11,697 (warfarin versus apixaban [8115], rivaroxaban versus apixaban [3582])	5.38 ± 1.50	4–5	0	NR
Kyriakoulis *et al*. (2024) [37]	Europe	37,811 (DOAC [3225], VKA [34,586])	NR	NR	NR	Systematic review and meta-analysis	35,797	28,654	NR	5	37,811	10,066 (26.63)
Chandra *et al*. (2023) [38]	India	176 (apixaban [88], warfarin [88])	45.32	Apixaban 63.58 ± 11.08, warfarin 61.76 ± 12.76	NR	RCT	NR	NR	Apixaban 4.77 ± 1.54, warfarin 4.71 ± 1.49	3 to 5	29	NR
Di Lullo *et al*. (2018) [39]	Italy	347 (warfarin [100], rivaroxaban [247])	44.67	Warfarin 66.5 ± 4.6, rivaroxaban 66 ± 4.4	16	Retrospective cohort study	240	122	NR	3b-4	NR	NR
Tscharre *et al*. (2024) [40]	Austria	383 (apixaban or rivaroxaban [218], VKA [165])	NR	NR	NR	Systematic review and meta-analysis of RCT’s	NR	NR	NR	5	383	NR
Ballegaard *et al*. (2024) [41]	Denmark	3208 (no anticoagulation [1833], anticoagulation [1375])	47.2	81 ± 10.37	NR	RCT	1.771	1.039	4 ± 1.48	5	NR	199 (6.2)
Coleman *et al*. (2019) [42]	USA	6744 (rivaroxaban [1896], warfarin [4848])	41.6	72 ± 63.8	NR	Retrospective cohort study	NR	NR	NR	4–5	NR	800 (11.9)
Elfar *et al*. (2022) [43]	Europe	33,516 (warfarin 30,472 [92.14 %], DOAC 3044 [8.91 %])	43.9	70.32 ± 4.6	NR	Systematic review and meta-analysis	34,272	33,796	4.28 ± 1.15	5	34,516	9591 (27.7)
Halperin *et al*. (2021) [44]	Canada	130	NR	NR	NR	Retrospective cohort study	NR	NR	NR	NR	NR	NR
Kim *et al*. (2021) [45]	Korea	89	46	66.4 ± 11.7	NR	Retrospective cohort study	88	44	4.4 ± 1.2	5	89	15 (16)
Laville *et al*. (2024) [46]	France	8954 (VKA [8471], DOAC [483])	37	73 ± 12.59	20 to 36	Retrospective cohort study	NR	4209	NR	5	8954	2060 (23)
Li *et al*. (2022) [47]	China	30,717 (NOAC [3744], warfarin [26,973])	NR	NR	NR	Systematic review and meta-analysis	NR	NR	NR	5	3744	NR
Mapili *et al*. (2023) [48]	Philippines	NR	NR	NR	NR	Systematic review and meta-analysis	NR	NR	NR	5	NR	NR
Navalha *et al*. (2024) [49]	Austria	517 (warfarin [341], DOAC [176])	NR	NR	5.71	Systematic review and meta-analysis of RCT’s	NR	129	NR	5	341	60 (17.6)
Park *et al*. (2023) [50]	Korea	260	43	70 ± 8.89	23 to 98	Retrospective cohort study	223	126	5 ± 2.22	4–5	NR	NR
Schafer *et al*. (2018) [51]	USA	604 (warfarin [302], apixaban [302])	50	Apixaban 73.5 ± 12.1, warfarin 70.6 ± 13.8	Apixaban 8.8, warfarin 9.7	Retrospective cohort study	482	280	4.8 ± 1.6	5	194	119 (19.7)
Shen *et al*. (2023) [52]	China	103,684	NR	60	NR	Systematic review and network meta-analysis	70%	70%	NR	5	72,579	162 (0.15)
Yang *et al*. (2023) [53]	China	6071	NR	NR	NR	Systematic review and meta-analysis	NR	NR	NR	5	NR	NR
Chen *et al*. (2021) [55]	China	1011 (DOAC [809], warfarin [202])	42.54	50	Warfarin (9 to 56), apixaban (8 to 67)	Retrospective cohort study	791	360	NR	4	16	NR
Pinner *et al*. (2022) [56]	USA	68 (VKA [36], NOAC [32])	54	67	NR	Retrospective cohort study	NR	NR	NR	5	68	NR
Moore *et al*. (2024) [54]	USA	110 (apixabane [53], VKA [57])	45.3	Apixaban: 68.74 ± 10.28, warfarin: 63.37 ± 16.18	Apixaban 24.50, warfarin 17.0	Retrospective cohort study	Apixaban: 2 ± 0.74, warfarin: 3 ± 2.96	NR	3 (both groups)	5	110	Apixaban: 32.1%, warfarin: 40.4%

SAH, systemic arterial hypertension; DM, diabetes mellitus; DOAC, direct oral anticoagulant; NOAC, non-vitamin K oral anticoagulant; VKA, vitamin K antagonist; RCT, randomized controlled trial; NR, not related; CKD, chronic kidney disease.

### 3.1 Major Bleeding

It is known that DOACs are associated with a lower incidence of major bleeding compared to warfarin in patients with AF and CKD. This is supported by several studies [25, 26].

Wetmore *et al*. (2022) [27] found that individuals on dialysis with non-valvular AF experienced fewer bleeding incidents while taking apixaban, regardless of dosage. Similarly, Reinecke *et al*. [30] confirmed these findings, demonstrating that apixaban led to fewer major bleeding events than the VKA phenprocoumon in hemodialysis patients. AlTurki *et al*. [31] further demonstrated that apixaban is safer than other anticoagulants for major bleeding events in individuals with AF and CKD stage G5.

De Vriese *et al*. [32] showed that rivaroxaban presented a lower risk of significant bleeding compared to VKAs in hemodialysis patients. Similarly, Chen *et al*. [34] found that rivaroxaban markedly reduced the risk of major bleeding in patients with AF and CKD stage G5. Yang *et al*. [53] further reinforced these findings by showing that apixaban is safer than warfarin in patients with non-valvular AF and CKD stage G5. In a hospital-based study, Pinner *et al*. [56] discovered that although warfarin increased the risk of major bleeding compared with DOACs, it effectively lowered the risk of thrombotic events. Moore *et al*. [54] also affirmed that apixaban has a superior safety profile for major bleeding compared to warfarin.

Kyriakoulis *et al*. [37] noted that patients treated with DOACs had a significantly lower risk of gastrointestinal bleeding than those treated with VKAs. Additionally, Chandra *et al*. [38] and Laville *et al*. [46] found that DOACs were less likely to cause bleeding, while remaining effective in preventing thromboembolic events. In contrast, Mapili *et al*. [48] and Navalha *et al*. [49] observed that while DOACs effectively prevented thromboembolic events, they did not significantly reduce the risk of major bleeding when compared to warfarin.

### 3.2 Stroke

Stroke prevention is the primary goal of anticoagulant treatment in patients with AF, particularly those with CKD, owing to the increased risk of thromboembolic events. Comparative studies on warfarin, DOACs, and non-anticoagulant strategies have yielded mixed results. One retrospective study suggested that VKAs may lower mortality from ischemic stroke; however, methodological limitations prevented definitive conclusions [27]. Research assessing the efficacy of oral anticoagulants in patients with AF on prolonged dialysis emphasizes the necessity of stroke prevention in this high-risk population. Additional studies have provided valuable insights into stroke prevention strategies in patients with moderate-to-advanced renal impairment.

An analysis comparing DOACs to warfarin in dialysis patients indicated that while DOACs are associated with an increased risk of major bleeding compared to conventional anticoagulants, they show similar rates of stroke and systemic embolism. This heightened risk of major bleeding with DOACs highlights the need for careful assessment when selecting the most suitable anticoagulant regimen for CKD patients.

These findings align with the existing literature, suggesting that DOACs offer a favorable safety profile with a reduced risk of stroke and systemic embolism. However, their use remains challenging due to the risk of major bleeding. Previous studies have underscored the complexity of managing anticoagulants in patients with renal impairment, emphasizing that the choice of anticoagulants must be tailored to the individual, balancing the need for stroke prevention with the risk of bleeding.

### 3.3 Mortality

Assessing the mortality associated with anticoagulant use in patients with CKD and AF is crucial for evaluating the benefits and risks of this therapy. Kim *et al*. [45] conducted a national survey, revealing that anticoagulant therapy can influence the survival of patients with AF and CKD on dialysis. Halperin *et al*. [44] and Coleman *et al*. [42] compared rivaroxaban with warfarin in these patients and provided important data on mortality. Additionally, Elfar *et al*. [43] conducted a meta-analysis that highlighted the need for more studies to assess the impact of different anticoagulants.

Fu *et al*. (2024) [36] evaluated the safety and efficacy of various oral anticoagulants (OACs) in patients with AF and advanced CKD at stages 4/5. They concluded that apixaban demonstrated a superior safety profile compared to warfarin and rivaroxaban. Similarly, Tscharre *et al*. (2024) [40] compared DOACs with VKAs in patients with non-valvular AF (NVAF) undergoing chronic hemodialysis and found no significant difference in total bleeding events, thromboembolic events, or overall mortality between DOACs and VKAs.

In three randomized controlled trials (RCTs) [26, 30, 32], the mean time in the therapeutic range (TTR) for patients in the VKA group was consistently reduced (50.7%, 44%, and 48%, respectively). These values are notably lower than those achieved in patients with some remaining renal function, despite frequent medical consultations in dialysis patients. The minimal risk of thrombosis was justified using non-vitamin K oral anticoagulants (NOACs) in this specific group of individuals.

The AXADIA-AFNET 8 study focused on significant safety events, with results that were consistent with previous trials comparing various anticoagulants [30]. Phase III studies of DOACs typically used stroke and systemic embolism as efficacy outcomes. In contrast, the AXADIA-AFNET 8 study employed a broader composite efficacy endpoint, encompassing cardiovascular mortality, stroke, myocardial infarction, pulmonary embolism, and deep vein thrombosis, reflecting outcomes highly relevant to patients and healthcare systems.

Three RCTs, including the present study, demonstrated that DOAC therapy is neither dangerous nor less effective than VKA treatment. Additionally, most observational studies involving patients with AF and stroke risk factors on dialysis who did not receive OACs also support the use of anticoagulants in this population [26, 30, 32].

### 3.4 Quality Assessment

The quality of the included studies was assessed using a structured framework specific to RCTs and observational studies. For RCTs, factors such as randomization methods, blinding, and completeness of follow-up were evaluated. Observational studies were examined for selection bias, confounding control, outcome measures, and statistical adjustment. Overall, the quality of the evidence varied. While RCTs typically offered more robust methodology, their findings were often limited by smaller sample sizes and the exclusion of dialysis patients. In contrast, observational studies provided broader insights into real-world effectiveness but were subject to biases such as confounding variables and incomplete data. The variation in study quality underscores the need for caution when interpreting results and highlights the importance of conducting high-quality trials to address unresolved questions.

## 4. Discussion

An analysis of 14 observational studies revealed several associations between VKAs and their clinical effects in patients on long-term dialysis [27, 28, 29, 36, 39, 42, 44, 45, 46, 50, 51, 54, 55, 56]. These studies found no significant reduction in thromboembolism risk with VKA use in this population. Additionally, low adherence to VKA therapy was a common concern. This review encompassing studies comparing VKAs with either no anticoagulation therapy or DOACs, indicated that VKAs did not significantly reduce thromboembolism but were associated with higher bleeding rates Table 2 (Ref. [25, 26, 27, 30, 31, 32, 34, 36, 40, 48, 49, 53, 54, 57]).

**Table 2.** **Category key findings supporting studies**.

Major bleeding	DOACs reduced major bleeding incidence compared to warfarin. Apixaban and rivaroxaban demonstrated lower risks of major and gastrointestinal bleeding. Some studies found no significant difference in bleeding risk between DOACs and VKAs.	Wetmore *et al*. (2022) [27], Reinecke *et al*. [30], AlTurki *et al*. [31], De Vriese *et al*. [32], Yang *et al*. [53], Mapili *et al*. [48], Navalha *et al*. [49]
Stroke	DOACs and VKAs show comparable stroke prevention. DOACs reduce systemic embolism risk but may increase major bleeding risk. Left atrial appendage occlusion (LAAO) devices are alternatives for high bleeding risk patients.	Harrington *et al*. (2023) [25], Pokorney *et al*. (2022) [26], Moore *et al*. (2024) [54], WATCHMAN device studies [57]
Mortality	DOACs, particularly apixaban and rivaroxaban, are linked to lower all-cause mortality than VKAs. RCTs and observational studies suggest comparable efficacy and safety, but some studies found no significant difference in mortality.	Fu *et al*. (2024) [36], Chen *et al*. (2021) [34], Tscharre *et al*. (2024) [40], AXADIA-AFNET 8 [30], RENAL-AF [26]

DOACs, direct oral anticoagulants; VKAs, vitamin K antagonists; RCTs, randomized controlled trials.

A network meta-analysis summarized the efficacy and safety of apixaban in patients across various dosing regimens, focusing on major bleeding, thromboembolism, and all-cause mortality. While dialysis and DOACs are now recognized as viable treatment options for patients with CKD, their concurrent use with renal replacement therapy, remains a topic of debate.

The AXADIA-AFNET 8 study found that the efficacy of DOACs was comparable to that of warfarin, and the RENAL-AF study reported no significant differences in the safety and effectiveness of apixaban versus VKAs [26, 30]. After one year, Kaplan-Meier estimates showed bleeding incidences of 32% for apixaban and 26% for VKAs. In RENAL-AF, apixaban was administered at doses of 5 mg and 2.5 mg twice daily, whereas the AXADIA-AFNET 8 exclusively used a 2.5 mg dose twice daily. Despite limited episodes preventing definitive conclusions, both studies reported an increased incidence of major bleeding. The minor differences in hemorrhagic episodes are likely attributed to random variability and dosing differences (2.5 mg vs. 5 mg apixaban). The pharmacodynamic data from RENAL-AF suggest that the 2.5 mg twice-daily dose tested in AXADIA-AFNET 8 achieves plasma concentrations similar to those found in patients without renal disease. In contrast, a twice-daily dose of 5 mg generated plasma concentrations similar to those observed in patients with CKD.

A trial involving 132 patients at three Belgian centers evaluated the efficacy of VKAs, specifically 10 mg rivaroxaban and 10 mg rivaroxaban combined with vitamin K2, at a 1:1:1 ratio [32]. The treatment groups were slightly smaller than those in the AXADIA AFNET 8 trial. Rivaroxaban was associated with significantly lower bleeding and thromboembolism rates, with hazard ratios for bleeding and thromboembolism of 0.39 and 0.41, respectively. The event rates per 100 patient-years for the primary efficacy outcome were 63.8, 26.2, and 21.4 in the VKA, 10 mg rivaroxaban, and 10 mg rivaroxaban plus vitamin K2 groups, respectively. In contrast, the VKA and apixaban groups had event rates of only 22.0 and 16.4, respectively.

Sarratt *et al*. (2017) [28] compared bleeding rates in patients with CKD stage G5 undergoing hemodialysis and found no significant differences between the apixaban and warfarin groups. In the Belgian trial, the incidence of death from any cause was higher in the VKA group, while the apixaban group had significantly lower event rates, potentially explaining rivaroxaban’s rivaroxaban over VKAs [32]. However, variations within the therapeutic time range may have influenced these results. Königsbrügge *et al*. (2017) [29] explored the prevalence of AF, application of antithrombotic therapies, and associated risk of thromboembolic events in patients undergoing hemodialysis. Their findings underscored the inherent challenge of preventing stroke in this population owing to the increased bleeding risk associated with CKD stage G5. This complex risk-benefit profile highlights the difficulties in achieving adequate anticoagulation while addressing safety concerns in such vulnerable patients.

The Food and Drug Administration (FDA) has approved the use of apixaban at a dosage of 5 mg twice daily for patients on dialysis, offering a potentially safer alternative to traditional therapies. However, it is important to note that most pivotal trials, including the ARISTOTLE study, excluded dialysis patients, resulting in limited evidence for this subgroup, which requires further investigation [58]. Meta-analyses, such as those conducted by Kuno *et al*. [35], suggest that 5 mg apixaban twice daily reduces mortality compared with warfarin or no anticoagulation, while being equally effective in preventing thromboembolism with a lower bleeding risk. Additional studies indicate that both 2.5 mg and 5 mg dosages of apixaban could provide comparable efficacy to warfarin, but with fewer adverse bleeding events.

Our analysis corroborates these observations, drawing on data from six studies [26, 27, 30, 31, 36, 38] on apixaban and one [32] on rivaroxaban. Although rivaroxaban demonstrated efficacy in reducing thromboembolism, its use was associated with higher rates of major bleeding than apixaban, suggesting a more favorable safety profile for the latter. Dabigatran showed a risk profile similar to apixaban, further validating the potential role of DOACs in this population. However, studies such as that by Kuno *et al*. (2020) [35] emphasize the limitations of DOACs, particularly dabigatran and rivaroxaban, in mitigating bleeding risks while preventing thromboembolism in dialysis patients.

Kao *et al*. (2024) [33] expanded on previous findings through a comprehensive meta-analysis incorporating nine additional cohort studies and data from the AXADIA-AFNET 8 trial [30]. This enriched analysis enabled subgroup analyses and categorization of therapeutic benefits by anticoagulant type. Notably, neither VKAs nor DOACs demonstrated consistent superiority in balancing bleeding and thromboembolism risks, raising questions about the necessity of anticoagulant therapy in long-term dialysis patients with AF. The lack of definitive evidence supporting AF as an independent risk factor for stroke in this population further complicates treatment decisions [58, 59, 60].

Shen *et al*. (2023) [52] proposed a protocol for the clinical use of DOACs in patients with CKD stage G5 and AF on dialysis. Although their recommendations emphasize individualized treatment strategies, they also highlight the need for robust evidence to ensure safety and efficacy. Similarly, Kao *et al*. (2024) [33] echoed these concerns, suggesting that without compelling evidence, withholding from anticoagulation may be a reasonable approach for selected dialysis patients with AF. This body of evidence illustrates the pressing need for further research to identify optimal anticoagulation strategies for patients with CKD stage G5 on dialysis. Future studies must prioritize rigorous trial designs that account for the nuanced risk profiles of this population, focusing on individualized approaches to minimize harm while maximizing therapeutic benefits. Addressing these gaps will provide clinicians with clearer guidance for managing anticoagulation in CKD stage G5 patients with AF.

Rivaroxaban demonstrated efficacy in reducing gastrointestinal bleeding and intracranial hemorrhage, whereas dabigatran should be used with caution due to an increased risk of major bleeding [52]. Park *et al*. (2023) [50] compared the safety and efficacy of DOACs with warfarin and no oral anticoagulation in patients with AF, advanced CKD, or CKD stage G5 on dialysis. The DOAC group had a significantly reduced risk of major or clinically relevant non-major bleeding compared to the warfarin group, and a lower risk of adverse clinical events compared to the OAC [50].

Future studies should aim to identify specific clinical characteristics to optimize the additional therapeutic advantages of apixaban, including its use in combined and split dosages. Concomitant anticoagulation therapy with dialysis is contraindicated in this population. Patients who received anticoagulant treatment were classified into two groups based on the HAS-BLED and CHA2DS2-VASc indices across all trials included in our analysis. However, randomized studies rarely incorporated these metrics or evaluated prothrombin time for safety and efficacy of VKAs. In contrast, various studies examining the risk of thrombosis and bleeding have produced contradictory findings regarding these characteristics. Furthermore, neither of the two randomized studies included patients categorized based on the aforementioned factors. Consequently, these patients did not exhibit any clinical characteristics, indicating the need for anticoagulants. Additional prospective studies are necessary to determine the specific conditions under which anticoagulants are administered.

The safety of anticoagulant use in patients undergoing dialysis for CKD stage G5 and AF remains a topic of ongoing debate, with comparative studies on warfarin, DOACs, and non-anticoagulant strategies yielding conflicting results. Historically, long-term dialysis patients have been treated with VKAs to prevent the thromboembolic complications associated with AF. A retrospective study indicated that VKAs may reduce the risk of ischemic stroke mortality [61]. However, the non-randomized nature of VKA studies limits the generalizability of their conclusions. Additionally, adjusting the VKA dosage based on prothrombin time is challenging because of altered metabolism due to uremia and concomitant heparin use in dialysis patients [53]. Harrington *et al*. (2023) [25] found the standard dosage of DOACs is safer and more effective than warfarin for patients with renal dysfunction, up to a creatinine clearance (CrCl) of at least 25 mL/min. Conversely, reduced DOAC dosages in patients with a CrCl of 25 mL/min were associated with higher risks of stroke, systemic embolism, and mortality without reducing the risk of major or intracranial hemorrhage. Similarly, Pokorney *et al*. (2022) [26] evaluated the safety and efficacy of apixaban in preventing stroke in patients with AF and CKD stage G5 undergoing hemodialysis. The study showed that apixaban had an incidence of 31.5% of relevant bleeding in one year compared to 25.5% in the warfarin group. Both groups had a low incidence of stroke [26].

Moore *et al*. (2024) [54] compared the efficacy and safety of apixaban and warfarin for stroke prevention in patients with NVAF and CKD stage G5 on hemodialysis. There were no statistically significant differences between apixaban and warfarin in terms of symptomatic, major, or minor bleeding [54].

Left atrial appendage occlusion (LAAO) devices have emerged as promising alternatives for stroke prevention in CKD stage G5 and AF. The WATCHMAN device, approved by the FDA in 2015, reduced both mortality and stroke rates in patients with AF. Comparative studies between OAC and LAAO in patients with CKD and CKD stage G5 revealed comparable procedural safety levels and significant reductions in stroke incidence [57]. LAAO devices have demonstrated safety and efficacy in patients with compromised renal function, which represents a promising area for future investigation. In CKD stage G5 patients with AF treated with warfarin, no significant reductions in ischemic stroke incidence were observed. Further research is needed to explore the potential of alternative anticoagulants, antithrombotic agents, antiplatelet agents, and LAAO devices in patients with AF and CKD stage G5 [62, 63]. Schafer *et al*. (2018) [51] demonstrated that, at 3 months, apixaban offers a comparable safety and efficacy profile to warfarin in patients with CKD stages 4 and 5, as well as those on dialysis, however bleeding rates did increase between 6−12 months. Therefore, apixaban is considered an acceptable alternative to warfarin in patients with severe renal dysfunction, although further studies are required to validate these findings [51].

The safety of anticoagulant use in dialysis patients with CKD stage G5 and AF remains a topic of debate. CKD is associated with a higher risk of blood clot formation [64], yet studies comparing warfarin, DOACs, and non-anticoagulant treatments in these patients have yielded conflicting results. Patients undergoing long-term dialysis have historically received VKAs to prevent blood clots associated with AF. A retrospective study suggested that VKAs could reduce ischemic stroke-related mortality [61]. However, the nonrandomized design limited consideration of confounding. Adjusting the VKA dosage based on prothrombin time is challenging because of the altered metabolism due to uremia and concomitant heparin use in dialysis patients [53]. Chen *et al*. (2021) [55] quantified the benefit-risk profiles of rivaroxaban and apixaban compared with warfarin in patients with NVAF and severe CKD or on dialysis. They found that rivaroxaban or apixaban use was associated with a significant reduction in the risk of gastrointestinal bleeding and all-cause mortality compared to warfarin use though further research is needed to confirm their safety and efficacy [55].

### 4.1 Future Perspectives

#### 4.1.1 Optimal Dosing

Studies should refine dosing strategies for DOACs, especially in dialysis patients, to maximize their efficacy while minimizing bleeding risk.

#### 4.1.2 Comparative Studies

To establish definitive recommendations, further RCTs are needed to compare DOACs, VKAs, and non-anticoagulant strategies in patients with CKD.

#### 4.1.3 New Treatments

Testing factor XI (FXI) inhibitors and novel anticoagulants offers new avenues for thrombosis prevention. Abelacimab (MAA868), an FXI monoclonal antibody, has shown promising efficacy and safety in clinical trials. A phase I study demonstrated that subcutaneous doses ranging from 5 to 240 mg/kg were well-tolerated and effective in both healthy and obese individuals. The ANT-005 TKA trial aimed to examine venous thromboembolism (VTE) prevention in total knee replacement patients, while the AZALEA-TIMI 71 study reported lower bleeding rates with abelacimab compared to rivaroxaban [65, 66, 67]. Early-phase trials have explored FXI inhibitors in patients with CKD stage G5, focusing on FXI’s primary role in thrombus development with minimal impact on hemostasis. These agents show potential for maintaining extracorporeal circuit patency during hemodialysis. A phase 2 trial investigated the pharmacokinetics, pharmacodynamics, and safety of IONIS-FXIRx in 49 hemodialysis patients [68, 69]. Similarly, a pilot phase 1 study is currently investigating the safety and tolerability of osocimab in patients undergoing hemodialysis. The Global Study of MK-2060 is an ongoing phase 2 trial examining the dosage of this anti-FXI monoclonal antibody in preventing arteriovenous graft thrombosis in patients with CKD stage G5 [70]. Abelacimab has the potential to significantly transform anticoagulant therapy by significantly reducing bleeding and thrombosis. Future research should focus on increasing the sample sizes, randomized comparisons of DOACs to VKA, and specific studies evaluating DOACs against emerging anticoagulants, including FDA-approved FXI inhibitors, in CKD stage G5 patients [71, 72].

### 4.2 Strengths

Our research has several notable strengths. First, the study involved an extensive literature review, incorporating evidence from RCTs, observational research, and meta-analyses. This comprehensive approach provided a thorough examination of anticoagulation treatments for patients with AF and CKD stage G5 undergoing dialysis. Second, we utilized a structured methodology guided by the PRISMA-ScR framework and Arksey and O’Malley’s five-stage scoping review process, enhancing the study’s methodological rigor, transparency, and reproducibility. Third, the investigation focused on a high-risk cohort of patients with CKD stage G5 on dialysis—a population frequently excluded from clinical trials. By comparing the safety and efficacy of DOACs with warfarin, this study addressed a critical gap in the existing literature. Fourth, the analysis provided valuable insights into key outcomes including severe bleeding, stroke prevention, and mortality rates. These findings are essential for making informed clinical decisions in AF patients with renal impairment. Finally, this research suggests future directions, such as exploring novel therapeutic approaches including factor XI inhibitors, which hold potential for improving anticoagulation treatment in this vulnerable patient population.

### 4.3 Limitations 

Our research has several limitations.

First, many clinical trials excluded patients with advanced CKD, necessitating reliance on observational studies, which may introduce biases. Second, the diversity in patient populations, DOAC dosing protocols, major bleeding definitions, and research methods, limits the generalizability of these findings. Third, a significant gap exists in the form of RCTs evaluating DOACs in patients with CKD requiring dialysis. Fourth, the use of varying DOAC dosing strategies across studies complicates the determination of optimal dosing regimens for dialysis patients. Fifth, small sample sizes in many studies, especially RCTs, limits the statistical power and raise concerns about the reliability of safety and efficacy conclusions regarding DOACs. Sixth, this review focuses primarily on apixaban and rivaroxaban, with insufficient information on other DOACs such as edoxaban, thereby narrowing the scope of the study. Seventh, some studies failed to stratify patients using clinical measures such as CHA2DS2-VASc and HAS-BLED scores, making it challenging to identify patients who would most benefit from specific anticoagulation strategies. Finally, while observational studies were included, the review lacks robust real-world evidence, particularly regarding the long-term outcomes of DOAC use in dialysis patients.

## 5. Conclusions 

While DOACs, particularly apixaban and rivaroxaban, demonstrate a favorable safety profile compared to warfarin, evidence remains inconsistent regarding the balance between thromboembolic prevention and bleeding risks in AF patients with CKD stage G5 or on dialysis. Future research should focus on optimizing dosing strategies and evaluating the long-term safety and efficacy of DOACs to improve anticoagulation management and clinical outcomes in this high-risk population.

## Supplementary Material

Supplementary material associated with this article can be found, in the online version, at https://doi.org/10.31083/RCM26736.
