# Supplementary material for: Anticoagulation Strategies for Atrial Fibrillation in CKD Stage G5 and Dialysis Patients: An Updated Scoping Review
Source: Rev Cardiovasc Med. 2025 Mar 5;26(3):26736. doi: 10.31083/RCM26736 (PMC11951491; doi:10.31083/RCM26736)
Supplement: Supplementary file 1 [file 2153-8174-26-3-26736-s1.zip › Supplementary Table 1.docx]

Supplementary Table 1. Summary of primary findings from selected studies.

| Author or study/Year | Objective | Major bleeding | Stroke | Mortality | Conclusion |
| --- | --- | --- | --- | --- | --- |
| Harrington *et al*. (2023) [25] | Perform a network meta-analysis to assess the safety and efficacy of DOACs and warfarin in patients with kidney function down to a CrCl of 25 mL/min, addressing concerns about using DOACs in this group. | The risk of S/SE, major bleeding, ICH, and death rose with declining kidney function. At a kidney function of 25 mL/min, the risk of major bleeding for patients on standard-dose DOACs was similar to those on warfarin (P interaction = 0.61). | Increase with declining kidney function. | Increase with declining kidney function. | When the CrCl level is at least 25 mL/min, standard-dose DOACs are both superior and safer than warfarin. Lower-dose DOACs in comparison, don't reduce bleeding or ICH risk, but do make S/SE and death more likely. The results show that standard-dose DOACs are superior to warfarin for patients with kidney problems. |
| Pokorney *et al*. (2022) [26] | To evaluate the safety and efficacy of apixaban in preventing strokes in patients with AF and ESRD on hemodialysis. | Bleeding rates: Major incidence or clinically relevant non-major bleeding: 31.5% (apixaban) vs. 25.5% (warfarin). Major bleeding: 11% (apixaban) vs 10% (warfarin), with one intracranial hemorrhage in each group. Most non-major bleeding occurred at hemodialysis access sites. | Both groups experienced reduced strokes, with apixaban at 3.0% and warfarin at 3.3%. | Death was the most common major event. In the apixaban arm, 26% of patients passed away, while in the warfarin arm, 18% of patients died. | Insufficient data prevented a definitive comparison of bleeding rates between apixaban and warfarin in AF patients with ESKD on hemodialysis. Bleeding events were 10-fold more frequent than strokes or systemic embolisms, underscoring the need for future studies to assess anticoagulation in these patients. |
| Wetmore *et al*. (2022) [27] | Evaluating dosage patterns of apixaban compared to warfarin in patients with NVAF receiving dialysis. | Standard and reduced doses of apixaban were associated with a lower risk of bleeding compared to warfarin. However, there was no significant difference in the risk of bleeding when comparing reduced apixaban dosage to standard apixaban dosage. | Apixaban may be a safer option for patients with CKD and AF to prevent stroke, as compared to warfarin. | In the analysis of mortality in patients receiving anticoagulation therapy, using the appropriate apixaban dosage was associated with a lower risk than warfarin (hazard ratio [HR], 0.85 [95% confidence interval [CI], 0.78–0.92]). | In patients with NVAF undergoing dialysis, it is crucial to consider that warfarin is linked to a greater risk of bleeding when compared with apixaban. |
| Sarratt *et al*. (2017) [28] | To compare bleeding rates in patients receiving apixaban or warfarin with ESRD undergoing chronic hemodialysis. | The warfarin group had seven major bleeding events, while the apixaban group had none (*p* = 0.34). Clinically relevant nonmajor bleeding rates were similar (12.5% vs 5.8%, *p* = 0.17), as were minor bleeding rates (2.5% vs 2.5%, *p* = 0.74). | NR | NR | There were no significant differences in bleeding rates between patients receiving apixaban and those receiving warfarin. Therefore, apixaban should be considered cautiously in hemodialysis patients until more is known about the effect of subsequent multiple doses on drug accumulation and clinical outcomes. |
| Königsbrügge *et al*. (2017) [29]  VIVALDI Study | To investigate the prevalence of AF and the use of antithrombotic therapy in patients on HD, as well as the risk of thromboembolic events in this population. | NR | This study recognizes that stroke prevention using antithrombotic agents is complicated by the excessive risk of hemorrhage in patients with ESRD on HD. | NR | Among HD patients, AF is commonly observed and is correlated with factors such as age, sex, and specific comorbidities. It is important to note that there is a lack of agreement on the best practices for stroke prevention in HD patients with AF. |
| Reinecke *et al*. (2023) [30] | Compare the effectiveness and safety of apixaban and the VKA phenprocoumon in AF patients undergoing chronic hemodialysis. | Primary safety events: major bleeding, non-major clinically relevant bleeding, or death from any cause, occurred in 45.8% of apixaban patients and 51.0% of VKA patients. | Primary efficacy events: ischemic stroke, all-cause death, myocardial infarction, and deep vein thrombosis or pulmonary embolism in 20.8% of apixaban patients and 30.6% of VKA patients. | Mortality rates: 16.2 per 100 patient-years (14.8 for apixaban; 17.6 for VKA. Cardiovascular mortality occurred in 13.4% (12 patients, (14.6% [7] for apixaban; 10.2% [5] for VKA). Total all-cause mortality was 22.7% (21, 18.8% [9] for apixaban and 24.5% [12] for VKA. | No significant differences in safety or efficacy outcomes were observed. Even on oral anticoagulation, patients with atrial fibrillation on hemodialysis remain at high risk of cardiovascular events. |
| AlTurki *et al*. (2024) [31] | To assess the safety and effectiveness of the anticoagulant apixaban versus warfarin in atrial fibrillation patients with ESRD requiring dialysis. | Apixaban significantly reduces severe bleeding, including intracranial and gastrointestinal, compared to warfarin. This is due to reduced renal excretion and monitoring requirements. | Apixaban effectively prevents strokes and systemic embolisms outperforming warfarin in AF patients with ESRD on dialysis. | NR | Observational studies show superior outcomes and fewer adverse events with apixaban compared to warfarin. |
| De Vriese *et al*. (2021) [32] | Comparing the effectiveness and safety of VKAs with rivaroxaban in AF hemodialysis patients. | Safety: rivaroxaban reduced major bleeding risk compared to VKAs. The rate of major bleeding was 18.2 per 100 person-years, considering a mean HAS-BLED score of five and a 28% history of gastrointestinal bleeding. | Effectiveness: fatal cardiovascular disease, nonfatal stroke, cardiac events, and other vascular events occurred in 35 VKA patients (rate of 63.8 per 100 person-years) AND 23 patients rivaroxaban (rate of 26.2 per 100 person-years). | Mortality: all-cause mortality was 30.6%, with no significant differences between groups. Primary causes of death were dialysis withdrawal, cardiovascular disease, and sudden death. | Rivaroxaban offers a superior risk-benefit profile compared to VKAs in AF patients undergoing HD. |
| Kao *et al*. (2024) [33] | The study unequivocally aims to establish the efficacy and safety of anticoagulation in dialysis-dependent patients with concomitant AF and ESKD, given the common association with a poor prognosis. | VKAs present a significantly higher risk of major bleeding compared to no anticoagulation or DOACs. | The efficacy of VKAs, DOACs, and no anticoagulation was similar in preventing thromboembolism. | NR | Dabigatran and rivaroxaban showed better efficacy for dialysis-dependent populations, while dabigatran and apixaban demonstrated improved safety. No anticoagulation was a non-inferior alternative, and VKAs were linked to the worst outcomes. |
| Chen *et al*. (2021) [34] | To compare the effectiveness and safety of DOACs and warfarin in patients with CKD who require anticoagulant therapy. | DOACs reduced significant bleeding risk by 17% compared to warfarin. Among Factor Xa inhibitors, apixaban showed the greatest risk reduction. | DOACs reduced the risk of stroke, systemic embolism, and venous thromboembolism (SE/VTE) by 22% compared to warfarin. Apixaban, a specific DOAC, showed an even more significant risk reduction of 25%. | NR | DOACs had better efficacy in early CKD and similar efficacy and safety to warfarin in CKD stages 4–5 or dialysis patients. |
| Kuno *et al*. (2020) [35] | This study explored the safety and effectiveness of oral contraceptive pills (OCPs) in patients with AF undergoing long-term dialysis. | Warfarin, dabigatran, and rivaroxaban pose a significantly higher risk of major bleeding when compared to apixaban and no anticoagulant. | DOACs did not show a significant reduction in the risk of stroke and/or systemic thromboembolism. | Apixaban 5 mg had a significantly lower mortality risk compared to warfarin, apixaban 2.5 mg, and no anticoagulant. | No evidence suggested that OACs reduce thromboembolism risk in AF long-term dialysis patients. Warfarin, dabigatran, and rivaroxaban were associated with a significantly higher risk of bleeding compared to apixaban and no anticoagulant. |
| Fu *et al*. (2024) [36] | Compare the safety and effectiveness of different oral anticoagulants in AF patients with advanced CKD stage 4/5. | Warfarin and rivaroxaban had higher rates of major bleeding than apixaban, showing apixaban's superior safety profile. | No significant differences found for ischemic stroke between the medications, but the bleeding rates were a key distinguishing factor. | All-cause mortality rates were similar among warfarin users (1.08; 0.98–1.18) and rivaroxaban users (0.94; 0.81–1.10) compared to apixaban. | Apixaban demonstrates a superior safety profile compared to warfarin and rivaroxaban. |
| Kyriakoulis *et al*. (2024) [37] | Examine the safety and effectiveness of DOACs versus VKAs in AF patients with ESRD on chronic hemodialysis. | Patients treated with DOACs had a significantly lower risk of gastrointestinal bleeding compared to those treated with VKAs in the general population. | The risk of ischemic stroke is comparable between patients treated with DOACs vs. VKAs. | Patients treated with DOACs had a comparable risk of all-cause mortality to those treated with VKAs. | Patients with AF and ESRD undergoing chronic hemodialysis DOACs and VKAs showed a similar risk of ischemic stroke, ischemic cerebral or systemic embolism, minor bleeding, major bleeding, and all-cause mortality. |
| Chandra *et al*. (2023) [38] | Comparison of the effectiveness and safety of apixaban and warfarin in AF patients with stage 3-5 CKD and assessment of the relative risks of stroke, thromboembolism, and major bleeding between the two groups. | Patients on apixaban had less major bleeding compared to those on warfarin after 12 months. | Apixaban increases time in the therapeutic range and reduces stroke risk in advanced chronic kidney disease patients. | NR | Patients with atrial fibrillation had a lower risk of stroke or thrombosis with apixaban compared to warfarin. This benefit extended to those with stage 4 and stage 5 CKD. |
| Di Lullo *et al*. (2018) [39] | Comparing rivaroxaban to warfarin in a significant cohort of stage 3b-4 CKD patients to assess the risk-to-benefit profile. | Warfarin may harm kidney function, while DOACs help preserve it and lower the risk of bleeding. | It is essential to prioritize DOACs over warfarin for patients with ESRD and AF due to the significantly lower risk of thromboembolic complications. | Patients with ESRD and an eGFR below 15 have a nearly 6-fold higher risk of mortality than those with an eGFR above 60. | Patients with CKD, especially those with ESRD who are already undergoing renal replacement therapy and require long-term anticoagulant treatment, present a significant challenge due to the heightened risk from anticoagulation adverse effects. |
| Tscharre *et al*. (2024) [40] | Compare the use of DOACs to VKAs in NVAF patients undergoing chronic hemodialysis. | No significant difference between DOACs and VKAs in total bleeding events (OR, 0.99; 95% CI, 0.63–1.54; *p* = 0.96). No significant difference for severe or life-threatening bleeding (OR, 0.65; 95% CI, 0.32–1.33; *p* = 0.24). | Although there was a trend towards a reduction in thromboembolic events or stroke in patients receiving DOACs, the change was not statistically significant. | There was no significant difference in overall mortality between patients treated with DOACs and VKAs. | Orally administered activated factor X inhibitors pose an equivalent risk of bleeding and death as AVKs in NVAF patients undergoing chronic hemodialysis. |
| Ballegaard *et al*. (2024) [41] | The use, effectiveness, and safety of OAC in AF patients with an eGFR of <30 mL/min/1.73 m^2^ undergoing dialysis. | Oral anticoagulation significantly increased major bleeding risk compared to no treatment. Major bleeding rates at one year: 7.6% (untreated patients) vs. 10.5% (oral anticoagulation). | Oral anticoagulation reduced the risk of thromboembolic events and death within one year. One-year risk: 3.6% (OAC) vs. 4.8% (no treatment). | Treatment with oral anticoagulation significantly reduces the risk of death. | For one year, OAC notably decreased the risk of a thromboembolic event while effectively mitigating the heightened risk of significant bleeding. |
| Coleman *et al*. (2019) [42] | To assess the efficacy and safety of rivaroxaban compared to warfarin for patients with NVAF and stage 4 or 5 CKD or those undergoing hemodialysis in routine practice. | Rivaroxaban had a 32% lower risk of major bleeding than warfarin in patients with NVAF and stage 4 or 5 CKD or undergoing hemodialysis. | NR | NR | When treating patients with NVAF and stage 4 or 5 CKD or those undergoing hemodialysis, it seems that rivaroxaban is associated with significantly lower rates of bleeding when compared to warfarin. |
| Elfar *et al*. (2022) [43] | To assess the safety and efficacy of DOACs vs. warfarin in AF patients with ESRD undergoing hemodialysis. | Compared to warfarin, DOACs were found to be linked to higher rates of minor bleeding. | In dialysis patients with AF, DOACs are just as effective as warfarin in preventing stroke and hemorrhagic stroke. | In dialysis patients requiring anticoagulation for AF, warfarin significantly reduced death rates compared to DOACs. | The study concluded that warfarin may provide greater benefits in reducing minor bleeding, systemic embolization, and mortality in hemodialysis patients with AF compared to DOACs. |
| Halperin *et al*. (2021) [44] | To assess perspectives and approaches in the use of anticoagulants for patients with atrial fibrillation undergoing dialysis. | NR | NR | NR | Clinical trials and consensus guidelines are needed to address the lack of comparative data and uncertainty in clinical practice. |
| Kim *et al*. (2021) [45] | To analyze the risks and benefits of anticoagulation in dialysis patients with AF. | Anticoagulant treatment, compared to patients not receiving treatment, increases severe bleeding (HR, 4.67; 95% CI, 1.26–17.25) and any bleeding (HR, 2.79; 95% CI, 1.01–7. 74). | NR | All-cause mortality and bleeding were not significantly different between no anticoagulation and apixaban treatment patients. | In dialysis patients with AF, anticoagulation therapy is associated with an increased incidence of severe bleeding. Still, anticoagulation therapy is associated with a low incidence of all-cause mortality and bleeding. cause mortality. Individualized anticoagulation therapy with careful bleeding monitoring is needed in dialysis patients with AF. |
| Laville *et al*. (2024) [46] | Compare to Efficacy and Safety of Off-Label Use of DOAC Versus Approved VKA. | The risk of major bleeding was numerically lower in patients treated with DOACs than VKA, but this difference was not statistically significant. | Off-label of DOACs use reduced thromboembolic event risk (ischemic stroke, systemic arterial embolism, systemic venous embolism, myocardial infarction, and unstable angina) compared to VKAs. However, no significant difference in efficacy was observed. | NR | In a large group of dialysis patients initiating an oral anticoagulant, the off-label use of DOACs was associated with a significantly lower risk of thromboembolic events and a non-significantly lower risk of bleeding relative to VKA use. This provides reassurance regarding the off-label use of DOACs in people on dialysis. |
| Li *et al*. (2022) [47] | Effect of NOACs vs. warfarin in AF patients undergoing dialysis. | DOACs showed an increase in the total risk of serious bleeding compared to conventional anticoagulants. | The use of NOACs showed similar incidences of stroke and SSE compared with warfarin. Factor Xa inhibitors did not significantly alter the risk of SSE compared with warfarin. | NR | Compared with warfarin, the use of NOACs, especially factor Xa inhibitors (rivaroxaban or apixaban), showed at least similar effectiveness and safety outcomes in AF patients on dialysis. |
| Mapili *et al*. (2023) [48] | Evaluate the safety and efficacy of DOACs compared with VKA in patients with CKD on dialysis and NVAF | There was no significant difference in the risk of major bleeding between DOACs and VKA (RR = 0.81, 95% CI, 0.46– 1.43). | The efficacy of DOACs was similar to that of VKA in patients with CKD on dialysis and NVAF. | Cardiovascular mortality was comparable between the two types of anticoagulants. | DOACs showed no significant difference in the risk of major bleeding, ischemic stroke, or cardiovascular death. Larger clinical trials are needed for validation. |
| Navalha *et al*. (2024) [49] | To evaluate the safety and efficacy of DOACs compared to VKAs in patients with AF on chronic hemodialysis. | The incidence of serious or life-threatening bleeding was similar between patients on DOACs and VKAs. | There was no significant difference between groups regarding ischemic stroke or transient ischemic attack (RR = 0.50; 95% CI, 0.19–1.35; *p* = 0.17). | There was no significant difference in cardiovascular mortality (RR = 1.34; 95% CI, 0.69–2.60; *p* = 0.39). | No significant differences were observed between DOACs and VKAs in cardiovascular mortality, all-cause mortality, ischemic/uncertain type of stroke or transient ischemic attack, or major stroke or life-threatening bleeding in patients with AF on chronic hemodialysis. |
| Park *et al*. (2023) [50] | Examine the safety and comparative effectiveness of DOACs compared to warfarin or no OAC in patients with AF and advanced CKD or ESRD on dialysis. | The DOAC group had a significantly reduced risk of major or clinically relevant non-major bleeding (CRNM) compared with the warfarin group (HR, 0.11, 95% CI, 0.01–0 .93, *p* = 0.043). | The DOAC group had a lower risk of composite adverse clinical events compared with the non-OAC group (HR, 0.16, 95% CI, 0.03–0.91, *p* = 0.039). | NR | Among AF patients with advanced CKD or ESRD on dialysis, DOAC was associated with a lower risk of primary or CRNM bleeding compared to warfarin and a lower risk of composite adverse clinical outcomes compared to no OAC. |
| Schafer *et al*. (2018) [51] | To evaluate major bleeding, stroke, and thromboembolism rates in patients with CKD stage 4, stage 5, and dialysis on apixaban or warfarin therapy. | Primary outcome: the occurrence of major bleeding 3 months after enrollment. Secondary outcomes: occurrence of major bleeding, ischemic stroke, and recurrence of VTE in 3 to 6 and 6 to 12 months | Apixaban may be an acceptable alternative to warfarin in patients with severe renal dysfunction. Future studies are for validation. | There were no differences in the rates of ischemic stroke or recurrent thromboembolism at any period. | Patients with advanced CKD taking apixaban had similar bleeding rates at 3 months compared with those taking warfarin. However, those who continued therapy had higher bleeding rates with warfarin between 6 and 12 months. |
| Shen *et al*. (2023) [52] | Evaluate the existing data and propose a practical protocol for the clinical utilization of DOACs in ESRD patients with AF undergoing dialysis. | Rivaroxaban: demonstrated efficacy in reducing gastrointestinal bleeding and intracranial hemorrhage. Dabigatran: caution due serious bleeding risk. Warfarin: Effective in reducing minor bleeding but does not offer significant protection against gastrointestinal or intracranial bleeding. | DOACs (rivaroxaban, apixaban, and dabigatran) prevent ischemic and hemorrhagic strokes. Rivaroxaban reduces ischemic stroke risk, dabigatran is recommended for preventing hemorrhagic stroke. Warfarin is not recommended for this purpose. | Apixaban, rivaroxaban and warfarin did not significantly reduce mortality compared to placebo. Rivaroxaban showed efficacy in reducing mortality, while warfarin and apixaban were not recommended for reducing mortality. Rivaroxaban was most effective in managing mortality, followed by warfarin and apixaban. | In conclusion, rivaroxaban demonstrated efficacy in reducing mortality and the incidence of ischemic stroke, gastrointestinal bleeding, and intracranial hemorrhage. Dabigatran is recommended for the prevention of hemorrhagic stroke. |
| Yang *et al*. (2023) [53] | Perform a systematic review and meta-analysis of the application of rivaroxaban in patients with NVAF and ESKD. | The study compared rivaroxaban with warfarin and apixaban, noting that rivaroxaban had a comparable risk of major bleeding to these controls. | No statistically significant differences in thrombotic events were observed between rivaroxaban and control. | NR | In this study, low-dose rivaroxaban (10 mg, once a day) may provide superior benefits to warfarin in patients with NVAF and ESKD. |
| Moore *et al*. (2024) [54] | To compare the efficacy and safety of apixaban and warfarin for stroke prevention in patients with NVAF and ESRD on HD. | There were no statistically significant differences between apixaban and warfarin in symptomatic bleeding, major or minor bleeding. A non-significant trend suggested major bleeding was more frequently in the warfarin group, with 52.4% versus 49.2% in the apixaban group. | NR | About 50.9% of patients in the apixaban group and 43.9% in the warfarin group died during the study period. Death was the most common reason for therapy discontinuation in both groups. | There were no statistically significant differences in efficacy and safety results between apixaban and warfarin in patients with NVAF and ESRD undergoing HD. |
| Chen *et al*. (2021) [55] | Quantify the benefit-risk profiles of the use of rivaroxaban or apixaban compared to warfarin in patients with non-valvular atrial fibrillation and severe CKD or on dialysis. | Rivaroxaban or apixaban use was associated with a significant reduction in gastrointestinal bleeding risk compared to warfarin. No significant differences intracranial bleeding risks were observed between rivaroxaban/apixaban and warfarin. The use of rivaroxaban or apixaban showed no significant differences in major bleeding risk compared to warfarin. | Rivaroxaban or apixaban use did not significantly reduce stroke or systemic embolism risks. There were no significant differences in ischemic stroke risks between NOACs and warfarin groups. | The use of rivaroxaban or apixaban was associated with a significant reduction in the risk of all-cause death (HR, 0.82, 95% CI, 0.72–0.93) compared with warfarin. | Current evidence suggests that rivaroxaban or apixaban are safe and at least as effective as warfarin in patients with AF and stage 4–5 CKD or on dialysis. |
| Pinner *et al*. (2022) [56] | This study evaluated the occurrence of major bleeding following the initiation of oral anticoagulation therapy in patients with ESKD in a community teaching hospital. | Occurrence of minor bleeding, thrombotic events, and hospitalizations due to bleeding or thrombosis. | NR | NR | Warfarin increased the risk of major bleeding in patients with ESKD compared with NOACs and did not reduce the risk of thrombotic events. |
| Moore *et al*. (2024) [57] | Compare the efficacy and safety of apixaban and warfarin in patients with NVAF and ESRD on HD. | Apixaban: 42.9%, Warfarin: 52.4% (not significant) | Apixaban: 7.5%, Warfarin: 10.5% (not significant) | Apixaban: 50.9%, Warfarin: 43.9% (not significant) | There were no significant differences in efficacy and safety between apixaban and warfarin. Further studies needed to assess this population. |

AF, atrial fibrillation; DOACs, direct oral anticoagulants; ESKD, end-stage kidney disease; NOACs, non-vitamin K antagonists; HD, hemodialysis; NVAF, nonvalvular atrial fibrillation; OAC, oral anticoagulation; ICH, intracerebral hemorrhage; HR, hazard ratio; NR, not relevant; CKD, chronic kidney disease; VKA, vitamin K antagonist; S, stroke.
